# Surface-Enhanced Raman Scattering of Silicon Nanocrystals in a Silica Film

**DOI:** 10.1038/srep27027

**Published:** 2016-06-03

**Authors:** Sergei Novikov, Leonid Khriachtchev

**Affiliations:** 1 Department of Micro and Nanosciences, Aalto University, P.O. Box 3500, FI-02015, Finland; 2 Department of Chemistry, P.O. Box 55, FI-00014 University of Helsinki, Finland

## Abstract

Surface-enhanced Raman scattering (SERS) is an intriguing effect, efficiency of which depends on many factors and whose applicability to a given system is not obvious before the experiment. The motivation of the present work is to demonstrate the SERS effect on silicon nanocrystals (Si-nc) embedded in silica, the material of high technological importance. Using the Ag overlayer method, we have found the SERS effect for this material. The best result is obtained for Ag layers of a weight thickness of 12 nm, whose surface plasmons are in a resonance with the laser wavelength (488 nm). The enhancement obtained for the Raman signal from 3–4-nm Si-nc in a 40-nm SiO_*x*_ film is above 100. The SERS effect is about twice stronger for ultra-small Si-nc (~1 nm) and/or disordered silicon compared to Si-nc with sizes of 3–4 nm. The SERS measurements with an Ag overlayer allow detecting silicon crystallization for ultra-thin SiO_*x*_ films and/or for very low Si excess and suppress the Raman signal from the substrate and the photoluminescence of the film.

Many limitations of modern electronic devices may be overcome by implementing photonics into electronics^1^. Integration of silicon-based photonics with CMOS technology is a promising approach since it gives the opportunity to merge electronics and photonics in the same chip^2^,^3^. Many optical functions such as light sources, amplifiers, waveguides, modulators, memory, and detectors should be achieved in order to fulfil this integration^4^. An efficient Si-based light emitter is probably the biggest challenge in the field.

Silicon nanocrystals (Si-nc) embedded in silica constitute one of the promising photonic materials^3^,^4^,^5^. In particular, an optical gain and a huge Raman gain have been demonstrated for this material^6^,^7^. Si-nc in silica films are conventionally prepared by thermal annealing of silicon-rich silicon oxide (SiO_*x*_, *x* < 2) or SiO_*x*_/Si multilayers above 1000 °C 8,9,10,11,12,13. Although the formation of Si-nc is accompanied by an increase of photoluminescence (PL), the PL mechanism is a controversial subject, and a number of mechanisms have been proposed^5^,^13^.

The Raman spectrum is a unique fingerprint of the material, and this technique has been extensively used to study Si-nc in silica films. The position and the width of the Raman band of Si-nc provide a valuable information on their sizes, stress, and temperature^13^,^14^,^15^,^16^. A known problem of conventional Raman spectroscopy is a small cross-section of this process. The use of high laser power should be avoided because it can change the properties of the samples, especially in Raman microscopy. The weakness of signals is a serious limitation for Raman spectroscopy of Si-nc in silica because the analysis of small Si-nc densities and very thin films is practically very important. Moreover, the weak Raman signals of Si-nc often overlap with their PL spectrum and the Raman spectrum of the substrate.

Surface-enhanced Raman scattering (SERS) at a rough metal (usually silver or gold) surface is a method, which can strongly increase Raman signals mainly due to the electromagnetic field enhancement associated with surface plasmons^17^,^18^. Very high enhancement factors have been demonstrated and the measurements of single molecules adsorbed on metal films have been performed using this method^19^,^20^,^21^,^22^,^23^. Fabrication of SERS substrates with nanoscale features is a principal issue in the area^24^,^25^,^26^,^27^,^28^, and metal-coated Si nanostructures have been used for this purpose^29^,^30^,^31^,^32^. Another approach to SERS is to deposit a metal island film on a top of the sample (overlayer method)^33^,^34^,^35^,^36^,^37^,^38^. This method does not produce very high enhancement factors; however, it is particularly useful in studies of thin solid films. Most of the success of SERS research has been connected with studies of organic molecules whereas the studies of nanomaterials are relatively few (see Refs 39, 40, 41, 42, and references therein).

The SERS effect depends on many factors, and very different enhancement has been reported. In general, the applicability of SERS to a given system is not obvious before the experiment. The motivation of the present work is to study the SERS effect on Si-nc embedded in a silica film, which is important because of wide applicability of this material. Using the Ag-overlayer method and a standard Raman microscope, significant enhancement of Raman scattering from Si-nc is obtained for the thinnest investigated films.

## Results

**Figure 1:**
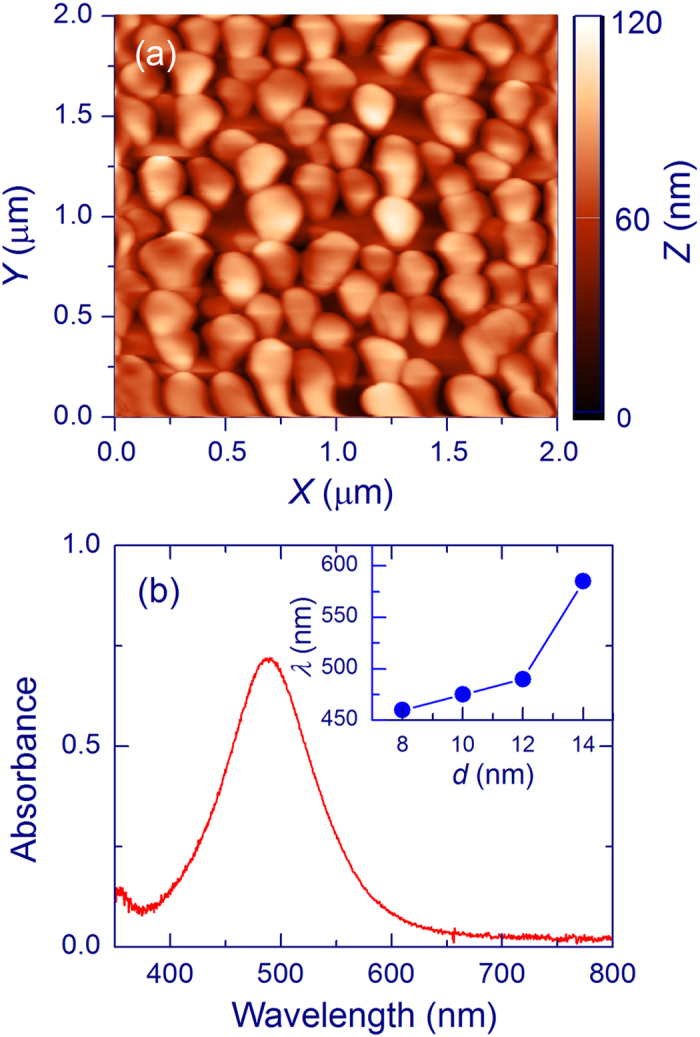
Characterization of Ag layers annealed at 400 °C. A typical AFM map (**a**) and absorption spectrum (**b**). The inset in panel b shows the position of the absorption maximum as a function of the Ag-layer weight thickness.

Ag layers annealed at 400 °C are constituted of islands with nanometer sizes (Fig. 1a). Qualitatively, the average size of the islands increases with the layer weight thickness. Fig. 1b shows a typical surface plasmon absorption spectrum of the Ag layers. The insert presents the position of the absorption maximum as a function of the Ag-layer weight thickness. The absorption maximum shifts to the red as the thickness increases. This dependence supports that the Ag layers contain nanoparticles, whose sizes increase with the weight thickness. These results are is general agreement with the literature data^18^,^43^,^44^,^45^,^46^. The SiO_*x*_ films annealed above 1000 °C show a Raman peak at ~517 cm^−1^, which originates from Si-nc with diameters of 3–4 nm^5^,^11^,^12^,^13^. A lower-frequency shoulder of this peak (≤510 cm^−1^) originates from smaller Si-nc and/or more disordered structures.

For the as-prepared SiO_*x*_ film (300 nm, annealed at 1100 °C), the use of an Ag overlayer with a weight thickness of 12 nm increases the 517 cm^−1^ Raman peak by a factor of about three-four compared with the uncoated film, which qualitatively indicates the SERS effect. In a test experiment, the initial film was thinned by 100 nm. Remarkably, the SERS signal of Si-nc from the thinner film (200 nm) appears larger by a factor of about five than that from the initial 300-nm film. Taking into account that SERS operates at very short distances^18^,^38^,^47^, this result suggests that annealing efficiently decreases the silicon content in the surface layer of a SiO_*x*_ film due to the oxidation of silicon by residual oxygen or by another mechanism. In fact, a decrease of the silicon concentration in a surface layer upon annealing was previously shown for similar samples by X-ray photoelectron spectroscopy^11^. Taking into account this depletion effect, we prepared a sample with film thicknesses of ~260, 190, 130, 80, and 40 nm, i.e. the thickest part was etched by ~40 nm. Even in this case, the SERS signal of Si-nc from the 260-nm film is somewhat smaller than that from the 190-nm film.

**Figure 2:**
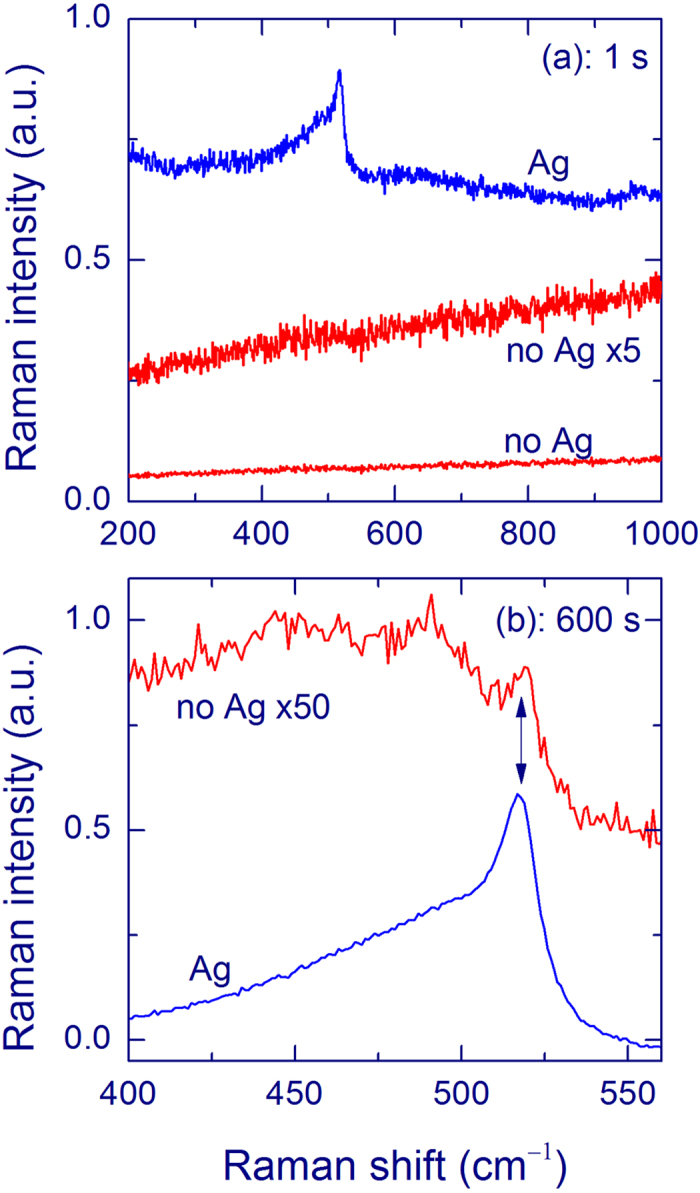
Raman spectra of a 40-nm SiO_*x*_ film annealed at 1100 °C measured using a 50× objective and 488-nm laser with exposures of 1 s (a) and 600 s (b). The red curves show the results for the uncoated SiO_*x*_ film (no Ag) and blue curves are for the SiO_*x*_ film with an Ag overlayer (Ag, weight thickness 12 nm). The Raman band at ~517 cm^−1^ originates from Si-nc with diameters of 3–4 nm. A broad band for the uncoated film is from the silica substrate. For the spectra in panel b, the linear background is subtracted.

Figure 2a shows the Raman spectra measured from a 40-nm-thick SiO_*x*_ film annealed at 1100 °C with an acquisition time of 1 s, excitation at 488 nm, and a 50× objective. Without an Ag overlayer, no signal of Si-nc (at ~517 cm^−1^) is observed (red curve). The slope of this curve is due to the PL of the annealed SiO_*x*_ film^5^,^11^,^12^,^13^. The spectrum changes drastically if it is measured with an Ag overlayer (weight thickness of 12 nm, blue curve). In fact, a strong Raman peak of Si-nc is observed at ~517 cm^−1^; thus, the SERS effect is significant.

For a longer acquisition time (600 s), a weak Raman peak of Si-nc at ~517 cm^−1^ can be detected from the 40-nm-thick SiO_*x*_ film without an Ag overlayer (red curve in Fig. 2b). In this case, a very broad and relatively strong Raman band in this region originates from the silica substrate. For the measurements with an Ag overlayer (weight thickness of 12 nm, blue curve in Fig. 2b), the Raman signal at ~517 cm^−1^ increases by a factor of ~120 compared with the uncovered SiO_*x*_ film, which gives the SERS enhancement, defined as the ratio of the Raman intensities with and without an Ag layer.

**Figure 3:**
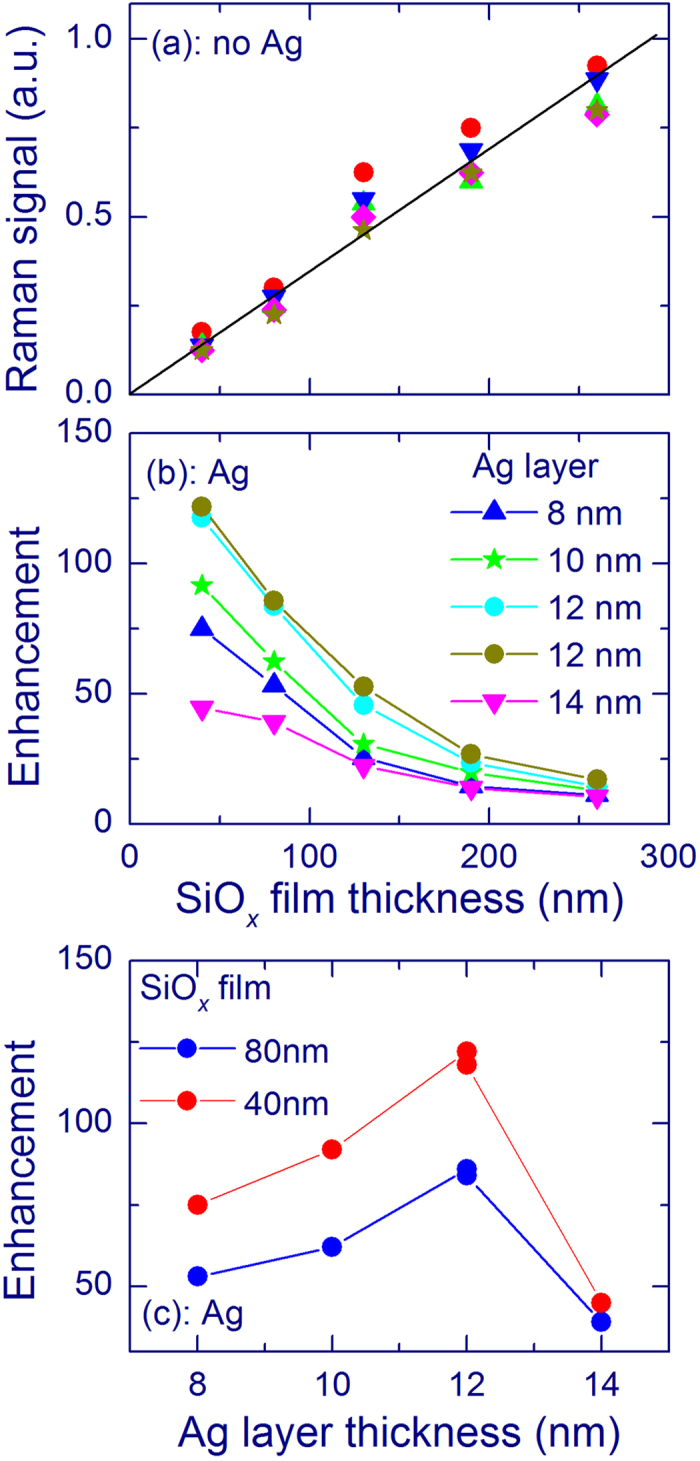
SERS effect for SiO_*x*_ films annealed at 1100 °C measured using a 50× objective and 488-nm laser with an exposure of 600 s. (**a**) Raman signal at 517 cm^−1^ of as a function of the thickness of uncoated SiO_*x*_ films (no Ag). Different symbols present the results of five experimental days. (**b**) SERS enhancement of the Raman signal at 517 cm^−1^ as a function of the SiO_*x*_ film thickness for different thicknesses of Ag overlayers. (**c**) SERS enhancement of the Raman signal at 517 cm^−1^ as a function of the Ag overlayer thickness for 40- and 80-nm-thick SiO_*x*_ films. The SERS enhancement is defined as the ratio of the Raman intensities with and without an Ag layer.

Different thicknesses of SiO_*x*_ films and Ag layers have been studied. The Raman signal of Si-nc of the uncovered SiO_*x*_ films (no Ag) is quite proportional to the SiO_*x*_ film thickness (Fig. 3a). The small deviations from the linearity can be due to inaccuracy of the thickness estimates and/or the interference of light in thin films. It is seen that the SERS enhancement of the Raman signal at ~517 cm^−1^ decreases as the SiO_*x*_ film thickness increases, similarly for all Ag layer thicknesses (Fig. 3b). Figure 3c shows the SERS enhancement as a function of the Ag-layer weight thickness. Two depositions of 12-nm-thick Ag layers led to very close results showing reproducibility of the structure with this thickness.

**Figure 4:**
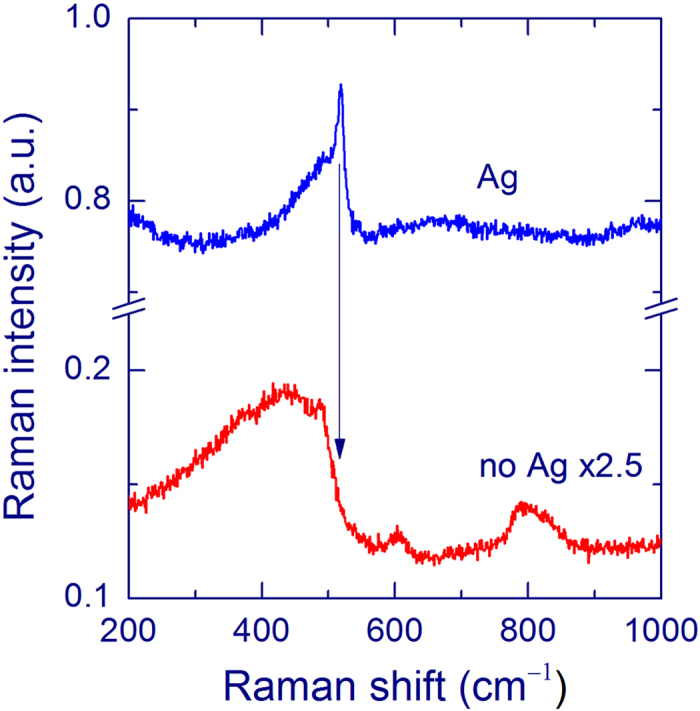
Raman spectra of a 40-nm SiO_*x*_ film annealed at 1100 °C measured using a 10× objective and 488-nm laser with an exposure of 60 s for the uncoated (no Ag, red curve) and Ag-coated (Ag, 12 nm, blue curve) SiO_*x*_ film. The Raman band at ~517 cm^−1^ originates from Si-nc with diameters of 3–4 nm. The broad bands measured for the uncoated film are from the silica substrate.

Figure 4 shows the Raman spectra measured with an objective 10×. Without an Ag layer, Raman scattering from the silica substrate dominates in the spectrum (red curve) and no band of Si-nc is observed. With an Ag layer, a Raman band of Si-nc appears whereas the substrate spectrum practically disappears (blue curve).

**Figure 5:**
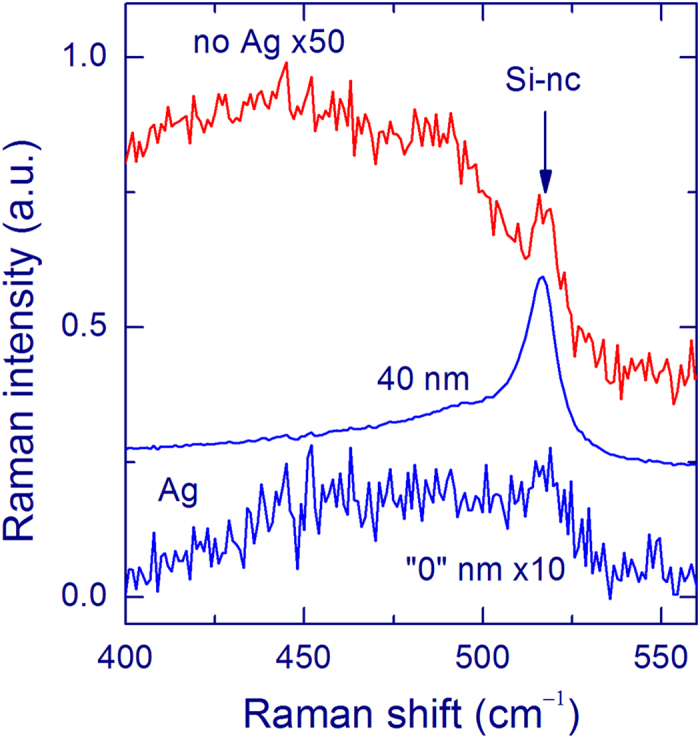
Raman spectra of a 40-nm SiO_*x*_ film annealed at 1200 °C measured using a 50× objective and 488-nm laser with an exposure of 600 s (two upper curves). The lowest curve corresponds to the sample area, for which the SiO_*x*_ film is practically etched (“0” nm). The red curve gives the results for the uncoated SiO_*x*_ film (no Ag) and blue curves are for an Ag overlayer (Ag, weight thickness 12 nm). The Raman band at ~517 cm^−1^ originates from Si-nc with diameters of 3–4 nm. A broad band for the uncoated film is from the silica substrate.

We studied the SERS spectra for SiO_*x*_ films annealed at a higher temperature of 1200 °C (Fig. 5). The SERS effect is also found for this annealing temperature, similar to annealing at 1100 °C. The lowest curve in Fig. 5 shows the spectrum measured with an Ag overlayer for a sample where the SiO_*x*_ film is supposed to be fully etched. However, it seems that minor traces of Si-nc are still present for this sample because an extremely weak band at ~517 cm^−1^ is still detectable. If an Ag overlayer is deposited on a silica plate, this peak does not appear.

**Figure 6:**
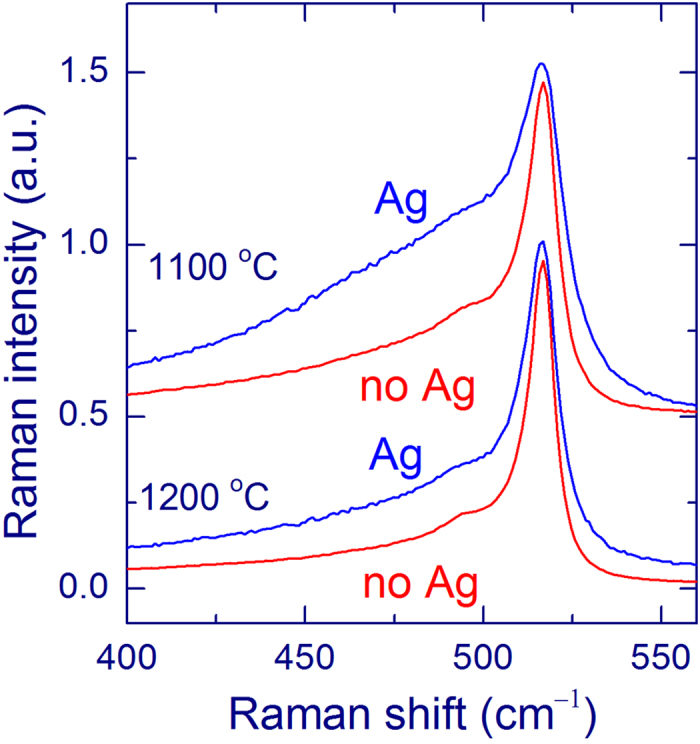
Raman spectra of SiO_*x*_ films annealed at 1100 and 1200 °C measured using a 50× objective and 488-nm laser with an exposure of 600 s. The red curves are for uncoated SiO_*x*_ films (no Ag) with a thickness of ~2 μm and the blue curves show the results for Ag-coated thin SiO_*x*_ films (Ag, weight thickness 12 nm). The Raman intensities at 517 cm^−1^ are equalized. The linear background is subtracted.

The Raman spectra were also measured from reference SiO_*x*_ films with relatively large thicknesses (~2 μm) annealed at 1100 and 1200 °C. These measurements allow the comparison of the shapes of the Raman spectra of Si-nc obtained with and without Ag overlayers and annealed at these two temperatures. It should be noticed that the Raman intensity at ≤500 cm^−1^ is also enhanced for the Ag-coated films. When the Raman intensities at 517 cm^−1^ are equalized (as in Fig. 6), the signal at ≤500 cm^−1^ is about twice larger for the Ag-coated films.

## Discussion

A strong Raman peak at ~517 cm^−1^ is observed from very thin SiO_*x*_ films for the measurements with an Ag overlayer, which qualitatively evidences the SERS effect (Fig. 2a). Raman bands with this shift are conventionally ascribed to Si-nc with sizes of 3–4 nm^5^,^12^,^13^,^14^. Weak peaks with this Raman shift are also observed from uncovered SiO_*x*_ films (Fig. 2b). The obtained enhancement of the Raman signal of Si-nc decreases as the SiO_*x*_ film thickness increases, similarly for all Ag-layer thicknesses (Fig. 3b). This trend is explained by the known fact that the SERS effect is short-range. Our experiments show that the SERS effect operates at distances below 40 nm for Si-nc in silica and the additional thickness does not increase the SERS signal significantly. In other words, the SERS enhancement originates from the part of the film, which is in a close contact with the Ag layer. The known theoretical and experimental estimates of this distance give for different molecular systems the operation distances of a few nanometers^18^,^38^,^47^.

The highest enhancement of the Raman signal of Si-nc is obtained for Ag layers with a weight thickness of 12 nm (Fig. 3c). It is remarkable that the plasmon absorption for this thickness is in a resonance with the laser wavelength (488 nm, Fig. 1b), which is an important factor for the SERS effect^18^,^41^,^48^. In fact, the SERS enhancement is found to be proportional to the Ag-layer absorbance at 488 nm. For the 40-nm SiO_*x*_ film and 12-nm Ag layer, the Raman intensity at 517 cm^−1^ is enhanced by a factor of ~120. However, it is highly probable that the effect would be higher for thinner SiO_*x*_ films. Similar measurements on SiO_*x*_ films with thickness of several nm cannot be reliable enough because the Raman signal from such uncovered SiO_*x*_ films (without Ag) would be extremely weak and the estimate of the film thickness is complicated.

Additional factors limiting the obtained SERS effect exist. Only a part of a SiO_*x*_ film is in a close contact with Ag nanoparticles (hot spots) as it is evident in Fig. 1a, which limits the average enhancement. The proportion of the SiO_*x*_ film surface in a close contact with Ag nanoparticles is difficult to estimate. In addition, it is possible that the etching efficiency of Si-nc is higher than that of the silica matrix. It would follow that Si-nc can survive after etching only below the film surface, which increases the average distance between Si-nc and Ag nanoparticles. Summarizing, we believe that the fundamental SERS effect on Si-nc is significantly stronger than we could demonstrate.

A broad Raman band around 480 cm^−1^ from the silica substrate is relatively strong for uncovered SiO_*x*_ films (Fig. 2b), which complicates the detection of a weak Si-nc signal from practical samples. The overlayer method leads to relative a suppression of the substrate signal providing an additional benefit for Raman spectroscopy of thin films. This effect is especially clear if an objective with a lower magnification is used, i.e. when the *Z* resolution is low. Figure 4 shows the spectra measured with an objective 10×. It is seen that the Raman spectrum of the silica substrate is practically absent in the SERS spectrum, in contrast to the spectrum of the uncovered film. The relative suppression of Raman scattering from the substrate is explained by absorption of light in an Ag layer. Indeed, the laser intensity is small in the substrate and the Raman scattering from the substrate is also attenuated by the Ag layer. Due to the spatial separation of the Ag layer and the substrate, the Raman intensity from the substrate is not enhanced. Figure 2a demonstrates that the PL from the SiO_*x*_ film is not enhanced by the Ag overlayer. This aspect can be important for samples with a low silicon excess, for which the PL is strong and the Raman signal of Si-nc is very weak^11^,^12^,^13^.

The band at ~517 cm^−1^ detected with an Ag overlayer originates from Si-nc with typical sizes of 3–4 nm^5^,^11^**−**13. It strongly decreases when the SiO_*x*_ film is almost completely etched out (Fig. 5). Moreover, this peak is invisible if an Ag overlayer is deposited on a silica plate. These facts support that this band originates from Si-nc in a silica matrix.

The Raman scattering at ≤500 cm^−1^ probably originates from ultra-small Si-nc with sizes of ≤1 nm^49^, although some fraction of disordered silicon is also probable^10^,^12^,^13^. It should be mentioned that this disordered silicon is different from amorphous silicon showing a broad Raman band around 470 cm^−1^ 12. For annealing at a higher temperature, the low-frequency Raman scattering decreases as measured with and without an Ag layer (Fig. 6). This trend is connected with the progressive crystallization of Si nanostructures^12^,^13^. In other words, the proportion of the ultra-small Si-nc decreases and the order increases as the annealing temperature increases. More remarkable is another observation. The Raman intensity at ≤500 cm^−1^ is also enhanced for the Ag-coated films relatively to the uncovered films. When the maxima of the intensities at 517 cm^−1^ are equalized (Fig. 6), the signal at 500 cm^−1^ is about twice higher for the Ag-coated films. This fact seems to suggest that the SERS effect is stronger for smaller Si-nc. As mentioned above, Si-nc may be efficiently removed near the film surface by plasma etching. In this situation, smaller Si-nc would be slightly closer to the Ag layer that the larger ones, which can contribute to the observed effect. This large sensitivity to ultra-small oxidized Si-nc is important because they are suggested to be the most optically active in this material^5^. Detection of broad Raman bands is very complicated in the presence of a strong background, and the present method considerably improves the situation.

In conclusion, we have demonstrated the SERS effect for Si-nc embedded in silica films using the Ag-overlayer method and a standard Raman microscope. The best results are produced by island Ag layers with a weight thickness of 12 nm whose surface plasmons are in a resonance with the laser wavelength (488 nm). For a 40-nm SiO_*x*_ film, the obtained enhancement is ~120 for the Raman intensity at 517 cm^−1^, which corresponds to Si-nc with sizes of 3–4 nm. The SERS effect is about twice stronger for scattering at ≤500 cm^−1^, originating from ultra-small Si-nc (≤1 nm) and/or from disordered silicon. Most probably, a significantly higher enhancement can be obtained for thinner SiO_*x*_ films and after correction for incomplete coverage of SiO_*x*_ films with Ag islands. In any case, the Ag-overlayer method makes possible to detect silicon crystallization for ultra-thin SiO_*x*_ films and/or for very low silicon excess, and it suppresses Raman signals from the substrate and PL of the SiO_*x*_ film. Moreover, due to the “shallow” sensitivity, it can provide depth profiles of the structures. We believe that this method can be successfully used on a routine basis to study a variety of nanomaterials.

## Methods

### Sample preparation

300-nm-thick SiO_*x*_ (*x* ~1.5) films were deposited on silica wafers by molecular beam deposition in a V-80M MBE system. The silicon flux was formed using evaporation of a silicon target by an electron gun (Leybold-Heraeus ESV-6 UHV). The atomic oxygen ambient used for silicon oxidation was generated by an RF plasma cell (Oxford Applied Research, MPD-21A). The samples were annealed in nitrogen atmosphere at 1100 and 1200 °C for 1 hour, which produced Si-SiO_2_ phase separation and Si crystallization^11^,^12^,^13^. The SiO_*x*_ films were thinned by plasma etching in an Oxford Plasmalab 80 RIE machine. A part of the surface was protected from the plasma by a photoresist layer. The thinning process was repeated several times decreasing the area protected by the photoresist. As a result, step-like profiles of the SiO_*x*_ films were obtained. Finally, the whole film was thinned by ~40 nm. Ag layers with weight thicknesses from 8 to 14 nm were deposited onto the SiO_*x*_ films by e-beam evaporation (Varian High Rate E-Gun system). A part of the sample surface remained uncoated with the help of a shadow mask and later it was used as a reference. Then, the samples with Ag layers were annealed at 400 °C for 1 h in nitrogen atmosphere, which made the Ag islands more separated from each other^45^. Reference SiO_*x*_ films with similar *x* and relatively large thickness (~2 μm) were also prepared and annealed in nitrogen atmosphere at 1100 and 1200 °C for 1 hour.

### Characterization

The heights of steps on SiO_*x*_ films were obtained with a profilemeter (Bruker, Dektak XT). The profiles of Ag layers were measured by an AFM microscope NT-MDT, NTEGRA. The micro-Raman spectra were recorded in a back-scattering scheme with a confocal Raman microscope (Horiba Jobin Yvon, LabRam HR 800) using excitation at 488 nm of an argon-ion laser (2.5 mW on the sample), 10× or 50× objectives, and spectral resolution of 2 cm^−1^. The fine adjustment of the distance between uncoated SiO_*x*_ films and the 50× objective was performed using the PL signal because the Raman signal of Si-nc was too weak for this purpose. The absorption spectra of Ag layers were obtained with a fiber-optics spectrometer (SD2000, Ocean Optics) and a broadband light source (Top Sensor Systems, DH-2000).

## Additional Information

**How to cite this article**: Novikov, S. and Khriachtchev, L. Surface-Enhanced Raman Scattering of Silicon Nanocrystals in a Silica Film. *Sci. Rep.* **6**, 27027; doi: 10.1038/srep27027 (2016).
